# Exosome and Macrophage Crosstalk in Sleep-Disordered Breathing-Induced Metabolic Dysfunction

**DOI:** 10.3390/ijms19113383

**Published:** 2018-10-29

**Authors:** Abdelnaby Khalyfa, Leila Kheirandish-Gozal, David Gozal

**Affiliations:** 1 Sections of Pediatric Sleep Medicine and Pediatric Pulmonology, Department of Pediatrics, Biological Sciences Division, The University of Chicago, Chicago, IL 60637, USA; 2 Department of Child Health and the Child Health Research Institute, University of Missouri School of Medicine, Columbia, MO 65201, USA; gozall@health.missouri.edu (L.K.-G.); gozald@health.missouri.edu (D.G.)

**Keywords:** exosomes, extracellular vesicles (EVs), macrophages, sleep-disordered breathing, obstructive sleep apnea (OSA) metabolic disorders, metabolic dysfunctions

## Abstract

Obstructive sleep apnea (OSA) is a highly prevalent worldwide public health problem that is characterized by repetitive upper airway collapse leading to intermittent hypoxia, pronounced negative intrathoracic pressures, and recurrent arousals resulting in sleep fragmentation. Obesity is a major risk factor of OSA and both of these two closely intertwined conditions result in increased sympathetic activity, oxidative stress, and chronic low-grade inflammation, which ultimately contribute, among other morbidities, to metabolic dysfunction, as reflected by visceral white adipose tissue (VWAT) insulin resistance (IR). Circulating extracellular vesicles (EVs), including exosomes, are released by most cell types and their cargos vary greatly and reflect underlying changes in cellular homeostasis. Thus, exosomes can provide insights into how cells and systems cope with physiological perturbations by virtue of the identity and abundance of miRNAs, mRNAs, proteins, and lipids that are packaged in the EVs cargo, and are secreted from the cells into bodily fluids under normal as well as diseased states. Accordingly, exosomes represent a novel pathway via which a cohort of biomolecules can travel long distances and result in the modulation of gene expression in selected and targeted recipient cells. For example, exosomes secreted from macrophages play a critical role in innate immunity and also initiate the adaptive immune response within specific metabolic tissues such as VWAT. Under normal conditions, phagocyte-derived exosomes represent a large portion of circulating EVs in blood, and carry a protective signature against IR that is altered when secreting cells are exposed to altered physiological conditions such as those elicited by OSA, leading to emergence of IR within VWAT compartment. Consequently, increased understanding of exosome biogenesis and biology should lead to development of new diagnostic biomarker assays and personalized therapeutic approaches. Here, the evidence on the major biological functions of macrophages and exosomes as pathophysiological effectors of OSA-induced metabolic dysfunction is discussed.

## 1. Sleep-Disordered Breathing

Obstructive sleep apnea (OSA) is the most common form of sleep-disordered breathing and is associated with many adverse health consequences, as well as with increased overall mortality risk [1,2,3,4,5]. OSA is characterized by repetitive obstructions of the upper airway during sleep that result in increased inspiratory efforts, sleep fragmentation (SF), and intermittent hypoxia (IH). OSA is particularly present among obese individuals and can affect at least 4–10% of all adults, with recent epidemiological studies reporting much higher prevalence [6,7,8]. Patients with OSA are at greater risk for metabolic dysfunction including insulin resistance, type 2 diabetes mellitus, dyslipidemia, and display evidence of adipose tissue inflammation and dysfunction [9,10,11,12,13,14,15]. Of note, both IH and SF have been independently associated with metabolic dysfunction, and acute exposures to IH were shown to decrease insulin sensitivity in healthy human volunteers [16,17]. In addition, studies in both rodents and humans have consistently reported that exposures to IH affects whole-body metabolic homeostasis, but the underlying mechanism(s) and the metabolic organs involved remain unclear [18,19,20,21,22,23,24,25,26,27].

## 2. Obesity, OSA and Metabolic Dysfunction

The worldwide prevalence of obesity has increased rapidly in the last 30 plus years in most Western countries. This increase has led to important changes in the pathogenesis and clinical presentation of many common diseases [28]. Obesity is considered a major social and health problem in both adults and children, and is viewed as a multifactorial disease caused by complex interactions between genetic and environmental factors. Obesity is manifest as increased abdominal adiposity and adipose tissue inflammation that are synergistically linked to an array of downstream health problems; including insulin resistance, type 2 diabetes, dyslipidemia, fatty liver disease, neurodegenerative disorders, and cardiovascular diseases [29,30]. Obesity is an important and major risk factor for OSA, with 30–64% of OSA patients being obese or overweight [6]. Among the negative behavioral factors that can facilitate the emergence of obesity, even in children, insufficient sleep due to either lifestyle associated short sleep duration and/or poor sleep quality are emerging as important and independent contributors [31,32]. Sleep disturbances can alter brain functions involved in the control of appetite, which can generate overeating in the current obesogenic environment, i.e., low physical activity and high availability of energy dense foods [33,34]. Obesity, in turn, significantly increases the risk of developing metabolic disorders, hypertension, stroke, and several types of cancer in up to 30% of obese patients, in addition to markedly increasing the risk of OSA, the latter being recognized as independently contributing to enhance the risk of such metabolic diseases and their deleterious consequences [35,36,37,38,39].

A hallmark feature of metabolically dysfunctional obese visceral white adipose tissues (VWAT) consists of the infiltration of immune cells, particularly macrophages [40,41,42,43]. Non-resident macrophages are traditionally classified into two types: pro-inflammatory (M1) and anti-inflammatory (M2), even if the distinction between these 2 sub-types is relatively artificial [44,45,46]. It is currently believed that altered polarization towards a pro-inflammatory M1 phenotype within VWAT is critically involved in cardiovascular and metabolic disease processes [43,47,48]. M1 macrophages express the surface marker CD11c [49,50].and produce pro-inflammatory cytokines, such as tumor necrosis factor α (TNF-α) and interferon γ (IFN) [51,52]. M2 macrophages express the surface marker CD301 and produce anti-inflammatory cytokines such as IL-10 [49]. The relative and absolute number of M1 macrophages increases in VWAT upon the emergence of obesity, thereby promoting adipose tissue inflammation [49]. In obese individuals adipocyte-derived exosomes have been identified as putatively contributing to the development of insulin resistance via activation of adipose-resident macrophages and the downstream secretion of pro-inflammatory cytokines that can foster the onset insulin resistance [53]. Furthermore, exosomes may carry pro-inflammatory factors via the circulation and interact with remote cell types to promote inflammation through activation of a variety of contributory pathways [54,55,56]. Here, we will critically review the extant published literature on macrophages and metabolic dysfunction in the context of obesity and diseases such as OSA, and further attempt to provide a mechanistic link implicating exosomes in such processes. 

## 3. Source of Macrophages

**Figure 1:**
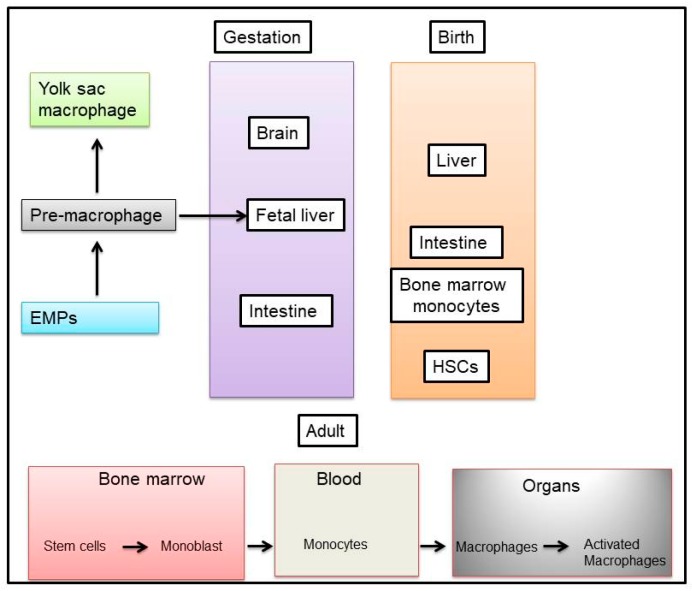
Sources of macrophages. Macrophages are distributed in tissues throughout the body and contribute to both homeostasis and disease. Erythro-Myeloid Progenitors (EMPs) is the source of pre-macrophages. Adult resident tissue macrophages originate during embryonic development rather than from circulating monocytes. During early gestation, macrophages are first observed and expand in the extraembryonic yolk sac during what is termed primitive hematopoiesis. At this stage in development, macrophages are the only ‘‘white blood cell’’ produced, because restricted progenitors in the yolk sac give rise only to macrophages and red blood cells. Later on, bone marrow derived cells will generate circulating monocytes and be independently recruited to the various organs and tissues.

Macrophages are recruited from precursor monocytes in the circulation, which in turn are derived from stem cells in the embryo and bone marrow [57]. Most tissue-resident macrophages are derived from embryonic precursors, however, under certain circumstances, circulating monocytes can differentiate into self-maintaining tissue-resident macrophages that resemble their embryonic counterparts [58]. Indeed, there are several markers that have been proposed to specifically identify macrophages of embryonic origin versus adult bone marrow monocyte-derived macrophages [58]. The first report about macrophage precursors, existing in the yolk sac and fetal liver of the early embryo, was published over four decades ago [59]. A few reports indicated how these cells contribute to various adult tissue-resident macrophage populations [60,61]. While macrophage origins clearly differ between organs, the origin of a tissue-resident macrophage does not seem to play a large role in determining its lifespan or functions [58,62], as illustrated in Figure 1. 

Macrophages that reside in adult healthy tissues are either derived from circulating monocytes or are established before birth, and are then maintained during adult life independently of monocytes [63]. It has been reported that most of the macrophages that accumulate at diseased sites typically derive from circulating monocytes. When a monocyte enters damaged tissue through the endothelium of a blood vessel, a process known as leukocyte extravasation, it undergoes a series of transformational and phenotypic changes to become a macrophage [60,64]. For example, embryonic macrophages were identified as F4/80^hi^CD11b^low-int^ cells, as opposed to F4/80^int-hi^ CD11b^hi^ macrophages, which are suggested to be of bone marrow origin; however, all of these precursors acquired a similar F4/80^hi^CD11b^int^ profile upon transfer into the alveolar space [62]. Furthermore, monocyte-derived Kupffer cells also acquired an F4/80^hi^CD11b^int^ profile that is equivalent to their embryonic counterparts [65]. In addition, *TNFRSF11a* was identified as a gene that is highly expressed by embryonic macrophages, but is only minimally expressed by hematopoietic stem cells (HSCs) and circulating monocytes. In Tnfrsf11a-Cre mice crossed with Rosa-YFP reporter mice [66], most tissue-resident macrophages (including alveolar macrophages and Langerhans cells) displayed a higher level of yellow fluorescent protein (YFP), labelling them as adult circulating monocytes [67], and therefore suggesting an almost pure embryonic origin of most tissue-resident macrophages. However, TNFRSF11a is highly expressed by both embryonically derived and monocyte-derived Kupffer cells, whereas it has low expression in alveolar macrophages regardless of origin [62,65]. Therefore, TNFRSF11a expression is not restricted to embryonic macrophages, and there is currently no reliable marker to accurately distinguish between macrophages of different origins. 

New evidence suggests that macrophages can originate from embryonic precursor cells that colonized developing tissues before birth (fetal tissue macrophages) and that tissue-resident macrophages have self-maintaining abilities in the adulthood. Murine animal models allowed the definition of three main sources for tissue-resident macrophages: (1) The yolk sac in the embryo as a source for progenitor cells by primitive hematopoiesis; (2) the fetal liver, where the hematopoiesis takes places, shifting from the yolk sac; and (3) the bone marrow that becomes the major hematopoietic center in late embryos and adult organisms [68,69,70]. Another scenario related to the model proposed that resident macrophages, developing in the embryo independently of the hematopoietic stem cell (HSC) compartment [71,72,73], still persist in adults, and can coexist with the so termed “passenger” leukocytes that include monocytes and DCs, which originated from bone marrow HSCs and myeloid progenitors [74], as shown in Figure 1. 

Macrophages are present in virtually all tissues, and differentiate from circulating peripheral blood mononuclear cells (PBMCs), which migrate into tissue in the steady state or in response to inflammation [75]. These PBMCs can develop from a common myeloid progenitor cell in bone marrow that is the precursor of many different cell types, including neutrophils, eosinophils, basophils, macrophages, dendritic cells (DCs), and mast cells. During monocyte development, myeloid progenitor cells (termed granulocyte/macrophage colony-forming units) sequentially give rise to monoblasts, pro-monocytes, and monocytes, which are released from the bone marrow into the bloodstream [75]. Monocytes migrate from the blood into tissues to replenish long-lived tissue-specific macrophages of the bone (osteoclasts), alveoli, central nervous system (microglial cells), connective tissue (histiocytes), gastrointestinal tract, liver (Kupffer cells), spleen, and peritoneum [75]. In blood, monocytes are not a homogeneous population of cells, and there is substantial debate about whether specific monocyte populations give rise to specific tissue macrophages [76]. In adults, monocytes originate from definitive hematopoietic stem cells (HSCs) through a characterized differentiation program involving progressively further committed progenitors. The identification of the monocyte-macrophage dendritic cell (DC) progenitor provided a developmental link between both DCs and monocytes within a common differentiation pathway [74]. While monocyte heterogeneity is not fully understood, one theory suggests that monocytes continue to develop and mature in the blood, while also being recruited to the tissues at various points during this maturation continuum [77]. The point at which they leave the blood may in fact define their function. In mice, two populations of monocytes from either end of this maturation spectrum have been identified and termed as “inflammatory” and “resident” monocytes, based primarily on the amount of time they spend in the blood before migrating into tissues [78].

## 4. Functional Aspects of Macrophages

Macrophages are formed through differentiation of monocytes, and are key players in the immune response, basically ridding the body of worn-out cells, foreign substances, microbes, in addition to cancer cells [79]. Since macrophages possess the ability to eliminate pathogens and of recruiting and instructing other immune cells, they play a central role in not only protecting the body, but also in contributing to the pathogenesis of inflammatory and degenerative diseases [80]. The mononuclear phagocyte system is particularly dynamic during inflammation or infection. Under physiological conditions, blood monocytes are recruited into the tissues, where they separate into macrophages. Depending on the microenvironment, macrophages can acquire distinct functional phenotypes [81]. It has been reported that monocytes/macrophages recruitment into a tumor microenvironment is mainly controlled by cytokines, chemokines, and growth factors produced by stromal and malignant cells [82]. 

Macrophages have been categorized into two artificially distinct activation states designated as classical (M1) and alternative (M2), even though there is a much wider spectrum of combinations of the two putatively distinct macrophage phenotypes [83]. For example, M1 activation occurs in response to molecules derived from bacterial infections such as lipopolysaccharide (LPS) and interferon-γ (IFN-γ). Highly inflammatory macrophages typically express the integrin α-chain Cd11c, CD11b, and F4/80 markers in mice, and are hence “triple” positive. M2 macrophages express CD11b and F4/80, but do not express Cd11c, hence “double” positive, and their phenotypic conversion usually occurs in response to parasites and their associated cytokines interleukin (IL)-4 and IL-13, promoting tissue repair and inhibiting M1 macrophages [84]. The majority of macrophages are located at strategic points and consequently each type of macrophage has a specific name (e.g., adipose tissue macrophages Kupffer cells in liver; alveolar macrophages in lungs; microglia in central nervous system; Hofbauer cells in placenta; intraglomerular mesangial cells in kidney); red pulp macrophages in spleen; epithelioid cells in granulomas; and osteoclasts in bone) [85]. 

**Figure 2:**
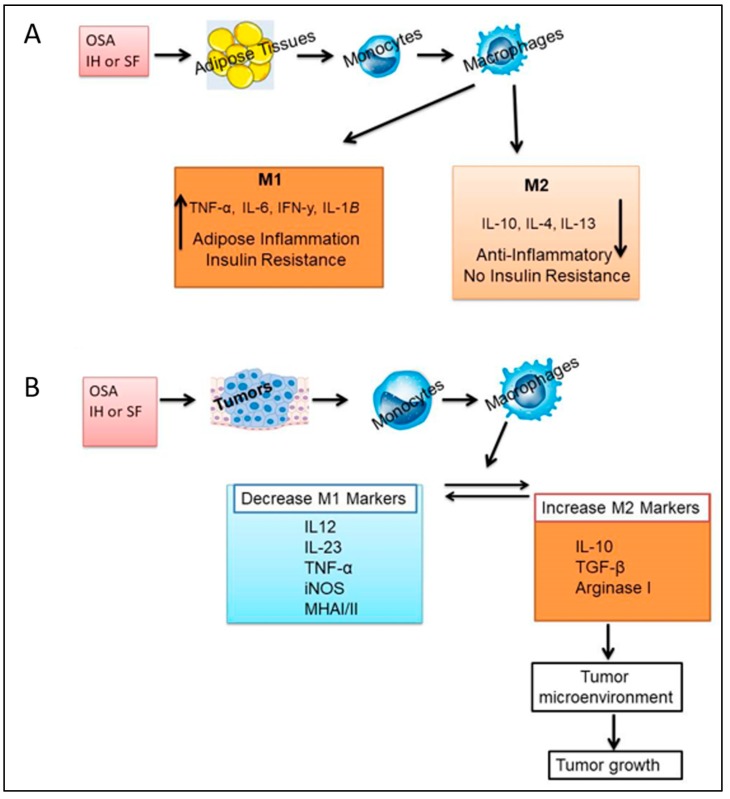
Effects of OSA on macrophages derived from adipose tissues and tumors. Tissue macrophages are composed of different subpopulations exerting different physiological properties. The two well-known subtypes are: M1 (classically activated macrophages) with pro-inflammatory properties, and M2 (alternatively activated macrophages) with anti-inflammatory properties. Macrophages are capable of dynamic inter-conversion depending on the immediate environment in which they evolve, and obesity or OSA increases tissue infiltration of macrophages and polarization towards the M1 phenotype (**A**). During tumor progression, circulating monocytes and macrophages are actively recruited into tumors where they alter the tumor microenvironment to accelerate tumor progression. The M1 macrophage is involved in the inflammatory response, pathogen clearance, and antitumor immunity. In contrast, the M2 macrophage influences an anti-inflammatory response, wound healing, and pro-tumorigenic properties (**B**).

In obese people, M1 and M2 macrophages induce tissue-specific metabolic responses such as hepatocyte biosynthesis of plasma proteins. They provide an early response to infection in the acute phase reaction, initiating features of systemic inflammation and infection such as loss of appetite as well as catabolism [86]. Another polarized macrophage phenotype which has been denominated tumor-associated macrophage (TAM) has been identified [87], and is often considered to be synonymous with M2 macrophages. While TAMs have some characteristics of M2 macrophages, they exhibit a transcriptional profile that is quite distinct from M1 and M2 [88], as shown in Figure 2A. Macrophages isolated from various tissues display remarkable differences in gene expression profiles, even though the circulating monocytes from which they originate are indistinguishable until they contribute to the macrophage pools in these tissues acquire these specific gene expression profiles [62,65,89,90,91]. 

In tumor microenvironment, macrophage polarization occurs through different ligands that modulate their metabolism, and macrophage plasticity is essential for the establishment of anti-tumor immune system functionality. These cells can vary from a configuration that inhibits tumor growth and induces cell death (M1 profile) to a configuration that stimulates cancer progression and tissue repair (M2 profile) [92], particularly important considering that macrophages are the most abundant immune cells in the tumor microenvironment [93]. 

The macrophage population phenotype can change occasionally as seen for example in obesity where there is a macrophage phenotype switch from M2 to M1 [94]. In contrast, tumor progression is often associated with macrophage phenotype changes from classically activated (M1) to alternatively activated (M2) [95]. There are several M2 sub-classifications. The M2a subtype is elicited by IL-4 or IL-13, the M2b subtype is elicited by IL-1 receptor ligands or exposure to immune complexes plus lipopolysaccharide (LPS), and the M2c subtype is elicited by anti-inflammatory stimuli, such as glucocorticoid hormones, IL-10, and transforming growth factor-β (TGF-β) [96,97] as shown in Figure 2B. 

Signaling and communication between endothelial cells and monocytes/macrophages play a critical role in cardiovascular homeostasis and the pathogenesis of atherosclerosis [98]. In addition to adipocyte-derived factors, increased release of tumor necrosis factor-α (TNF-α), interleukin-6 (IL-6), monocyte chemoattractant protein-1 (MCP-1), and additional products of macrophages and other cells that populate adipose tissue also plays a role in the development of cardiovascular and metabolic risk including insulin resistance [99,100]. As mentioned above, during obesity, macrophages accumulate in visceral white adipose tissue (vWAT), where they promote chronic low-grade inflammation. It is well established that this inflammation is causally associated with insulin resistance. Two models have been proposed to explain the increase in the number of M1 macrophages in vWAT upon obesity: (a) Circulating monocytes are recruited to vWAT, where they differentiate into M1 macrophages, and (b) obesity induces the proliferation of resident macrophages in vWAT [101,102]. 

## 5. Macrophage and Metabolic Dysfunction

Obesity and metabolic syndrome are becoming increasingly prevalent, and raise the risk of type 2-diabetes (T2D) cardiovascular diseases and cancer. Activation of leukocytes and inflammation of adipose tissue are established links between obesity and development of metabolic dysfunction [27,103]. It has been indicated that metabolic dysfunction and insulin resistance can shift the balance between numerous pro and anti-inflammatory regulators of macrophages, while also having the ability to create a feed-forward loop of increasing inflammatory macrophage activation. These processes ultimately worsen adipocyte dysfunction [41]. Adipose tissue macrophages (ATMs) are the predominant leukocyte in lean, metabolically healthy adipose tissues, and accumulate in obese individuals to constitute up to 40% of the stromal vascular cell fraction [104]. It has been reported that obesity-related changes in adipose tissue leukocytes, in particular ATMs and dendritic cells (ATDCs), are implicated in metabolic inflammation, insulin resistance, and altered regulation of adipocyte function [105,106,107,108].

As mentioned, OSA is a common condition across the life spectrum, and many cross-sectional and longitudinal studies have now clearly established OSA as an independent risk factor for the development of a variety of adverse metabolic disease states, including hypertension, insulin resistance, (T2D), nonalcoholic fatty liver disease, dyslipidemia, and atherosclerosis [109,110,111]. We have previously shown that mice exposed to long-term SF develop increased body weight and adipose tissue mass, along with mobilization and differentiation of adipocyte precursors, as well as adipose tissue inflammation [12,112,113,114]. This contrasts with mice exposed to chronic IH who display reductions in body weight, along with increased visceral fat inflammation [9,26,115,116]. However, despite their divergent effects on adipose tissues, these two hallmark characteristics of OSA induce metabolic dysfunction and insulin resistance, suggesting that in the context of OSA, excessive body weight may potentiate the effects of obesity. vWAT has emerged as an attractive effector of these adverse consequences, given the strong link between OSA and obesity. In obese individuals, adipocyte-derived exosomes have been implicated to the development of insulin resistance, via activation of adipose-resident macrophages and secretion of pro-inflammatory cytokines that can result in insulin resistance [53,117,118]. However, exosomes may also carry pro-inflammatory factors via the circulation and interact with remote cell types to promote inflammation through activation of contributory pathways [54,55,56]. ATMs play pivotal roles in the establishment of the chronic inflammatory state and metabolic dysfunctions such as T2D and IR [119,120]. In addition, either genetic or diet-induced adipocyte expansion promotes the accumulation of macrophages in vWAT in mice, and the majority of obese patients [51,121].

The interaction between adipocytes and macrophages aggravates the chronic inflammation in obese vWAT [122]. Furthermore, atherosclerosis is a chronic inflammatory disease driven by an imbalance in lipid metabolism and a maladaptive immune response [123]. In vivo, several studies have shown macrophage heterogeneity within the atherosclerotic plaque in response to the exposure to lipids and their oxidized derivatives [124]. de Gaetano et al. [125] observed a marked difference in a macrophage subset between symptomatic and asymptomatic plaque [125]. Moreover, in murine models, it has been demonstrated that in the regressing plaque a decrease in the number of macrophages occurs and, in some, a switch of their phenotypic characteristics occurs along with enrichment in M2-like phenotype, suggesting that this is a common signature of regressing plaques [126]. 

## 6. Exosome Biogenesis

Extracellular vesicles (EVs), are membrane-contained vesicles originating from the endocytic pathway or from the cell plasma membrane. They are released into the extracellular space by virtually all cells, playing an important role in intercellular communication during physiological and pathological processes. In particular, exosomes, a sub-class of EVs (30−120 nm), have generated considerable attention as intercellular signal transmitters [109,127]. Exosomes have been described in cell culture supernatants as well as plasma, serum, and virtually all bodily fluids [128,129], and serve as intercellular communication vehicles through delivery of proteins, lipids, nucleic acids, or other components in or within their lipid bilayer membrane to neighboring or distant cells. Exosome biogenesis consists of two steps, the inward budding of membranous vesicles of endosomes and their release into a structure known as a multi-vesicular body (MVB). The formation of MVBs occurs during the maturation of early endosomes into late endosomes with the accumulation of intraluminal vesicles [130,131]. After maturation, MVBs are directed for fusion with either the lysosome, where their cargo will undergo lysosomal degradation, or to the plasma membrane, where their contents will be released into the extracellular space [132]. 

## 7. Exosomes Isolation and Characterization

**Figure 3:**
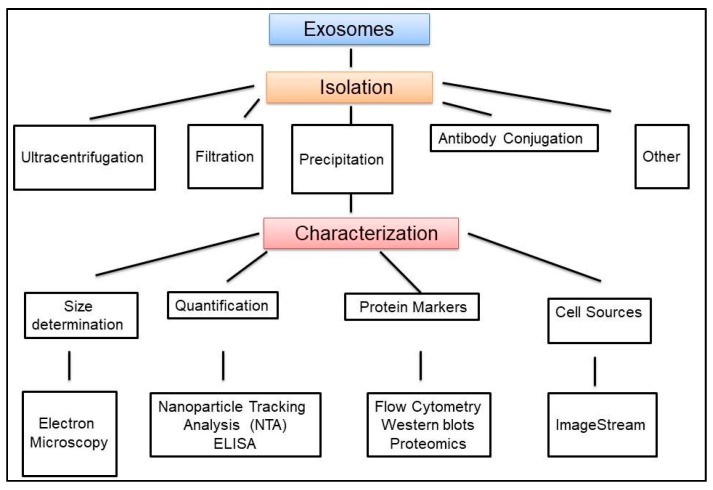
Exosome isolation and characterization general pipeline. Several methods are used for exosome isolation, and need to include validation and characterization of exosomes isolates.

Exosome isolation approaches are variable among different research groups and also depend on the sample source from which they are obtained [56,127,128]. Therefore, exosome isolation and purification need to be standardized to ensure that delineation of exosome populations and their functional analysis is reproducible and comparable across different research groups. Among the multitude of techniques for the isolation of exosomes, specific feature of exosomes are used, such as shape, density, size, and surface protein markers, as shown in Figure 3.

Ultracentrifugation methods use high speed centrifugation to pellet vesicles, while polymer-based reagents are added to the sample to facilitate vesicle precipitation using lower speeds. Ultrafiltration involves the concentration of vesicles from a large volume of biological fluid using a centrifugal filter unit. Size exclusion chromatography and density gradient separation are both designed to allow the separation of vesicles from other nonvesicular debris. Immunoaffinity capture methods use antibody-coated beads to selectively isolate vesicles displaying a surface marker of interest. Eventually, the choice of purification method for an individual experiment is influenced by time, cost, and equipment considerations, as well as the sample requirements for any downstream analyses. Regardless of which protocol is ultimately used, the cellular source(s) of exosomes must be identified. Both concentration and composition of exosomes can vary significantly during disease. Isolation and characterization of exosomes can provide important information for early disease detection, monitoring disease status, and the development of effective treatments. We should remark that determining the size distribution of exosomes is a critical step for exosomes studies. Size distribution measurement technologies include electron microscopy (EM), nanoparticle tracking analysis, resistive pulse sensing, and atomic force microscopy (AFM). Transmission EM (TEM) has been so far the preferred technique for direct observation of the size and morphology of exosomes [133]. A device allowing Nanoparticle Tracking Analysis (NTA) has been developed to measure the size distribution and concentration of nanoparticles [134]. More recently, faster and most sensitive antibody-based methods to quantify exosomes have been based on standard curves of known exosomes that include EXOELISA-ULTRA, EXOELISA, EXOCET, FLUOROCET provided by commercial sources (https://www.systembio.com; https://www.biovision.com) (Figure 3). 

## 8. Exosome Cargo

According to the most recent version of the exosome content database, ExoCarta (http://www.exocarta.org), exosomes from various organisms and various cell types have been characterized as potentially containing 9769 proteins, 1116 lipids, 3408 mRNAs, and 2838 miRNAs [135]. The protein content largely depends on the exosome cellular origin, and is generally enriched for certain molecules, including targeting and fusion proteins (e.g., tetraspanins, lactadherin, and integrins), cytoplasmic enzymes (e.g., GAPDH, peroxidases, pyruvate kinases, and lactate dehydrogenase), chaperones (e.g., heat shock proteins Hsp60, Hsp70, Hsp90, and the small HSPs), membrane trafficking proteins (e.g., Rab proteins, ARF GTPases, and annexins), proteins involved in MVB formation (e.g., ALIX, TSG101, and clathrin), cytoskeletal proteins (e.g., actin and tubulin), and signal transduction proteins (e.g., protein kinases and heterotrimeric G proteins) [129]. Several studies have shown that besides proteins, exosomes also carry certain types of lipids, which play an important role in maintaining the biological activity of exosomes [136,137]. 

In addition to proteins and lipids, exosomes also contain nucleic acids including mRNAs and other non-coding RNAs such as miRNAs and lncRNAs, and these exosomal RNAs, in particular the miRNAs have been shown to be functionally important in the recipient cells [138,139,140]. Mature miRNAs identify target mRNAs and regulate post-transcriptional gene expression [141]. Exosomes protect miRNAs from degradation induced by RNA enzymes in body fluids. They also transport miRNAs to recipient cells, where they participate in gene expression and signal transduction playing a key role in the processes of various diseases. There is growing evidence that the maturation process of miRNAs is linked to the formation and maturation of exosomes, and exosomal miRNAs play important roles in metabolic diseases, where they can be regarded as biomarkers and targets for correcting metabolism disturbances [142,143]. 

## 9. Exosome Internalization

While the important roles of exosomes in many physiological and pathological processes are being revealed, the mechanism of exosome-cell interactions remains unclear. Exosomes can deliver their cargo to recipient cells through direct interactions between the exosomes and the cell membrane. Cargo delivery was demonstrated by independent studies focusing on the exosomal shuttle of cell type specific or specie-specific proteins and RNAs. Direct visualization of stained exosomes by fluorescent microscopy has further provided evidence for exosome-cell interactions [56,144,145]. It is reported that the uptake of exosomes by target cells may occur through three different mechanisms: (i) Simple fusion of the exosome with the cellular membrane, directly releasing the content of vesicles into the cytoplasm; (ii) exosome uptake by endocytosis; and (iii) uptake dependent on the presence of distinct receptor proteins that enable binding of exosomes to target cells [146,147,148]. It is generally accepted that the cell of origin and secretion conditions of exosomes seem to determine their cell surface content, and consequently the cell-type-specific adhesion molecules, targeting exosomes to specific cells [149]. Nevertheless, exosomes contain many different cell surface molecules and one single exosome is able to engage many different cell receptors [149]. 

We and other have investigated cell uptake in several cell lines [150,151,152,153]. Several methods were used to study exosomal uptake in vitro and in vivo. For examples, exosomes were labeled using different commercially available dyes including PKH26 Red and PKH67 Green Fluorescent Cell Linker Kit for General Cell Membrane Labeling [152,154,155,156]. Treatment of exosomes with proteinase K significantly reduced uptake by ovarian cancer cells. These results indicate that surface proteins on exosomes may serve as receptors for uptake [157]. The uptake of tumor-derived exosomes seems to be mediated by surface phosphatidylserine, which can be blocked with diannexin [158]. Moreover, exosome internalization could be inhibited by the knockdown of dynamin 2, which is necessary for clathrin and caveolin-dependent endocytosis [159]. To improve exosome uptake, [160] we have proposed to combine cationic lipids and a pH-sensitive fusogenic-GALA peptide, increasing exosome binding at the plasma membrane, and improving uptake via the endocytic pathway. Interestingly, heparin blocks both binding to the membrane of recipient cells and uptake of glioblastoma-derived exosomes by human endothelial cells ([161].

## 10. Exosomal Function

The presence of exosomes in healthy body fluids advocates a role of these vesicles in the normal physiology of the body, including communication in the immune system, tissue repair, and communication within the nervous system [162,163]. Originally, exosomes were described as a mechanism for elimination of excessive proteins or undesirable molecules from the cell [164]. However, we now have evidence that exosomes control both normal physiological processes, such as immune responses and lactation [165], and the expansion and progression of diseases, such as neurodegenerative diseases and especially cancer [166,167]. Exosomes carry out a diverse range of functions and sometimes have opposing effects on the recipient cells depending on their tissue of origin and molecular content [168]. Thus, they may serve as invaluable biomarkers for disease diagnosis, prognosis, and therapy [166]. By delivering nucleic acids such as miRNAs or mRNAs to target cells, exosomes can exchange genetic information between cells. For example, exosomes from a mouse and a human mast cell line (MC/9 and HMC-1, respectively) and from primary bone marrow-derived mouse mast cells were shown to contain RNA [140]. In vitro studies showed that, after transfer of mouse exosomal RNA to human mast cells, new mouse proteins were detected in the recipient cells, indicating that transferred exosomal mRNA was translated after entering the cell. Additional studies supported exosomes-mediated functional delivery of mRNA [169] and small RNAs [144,170] to acceptor cells. Furthermore, exosomes derived from either the lung or liver entered bone marrow cells in vitro and induced expression of proteins specific for the originating lung or liver tissues [171]. These findings indicate that exosomes have the capacity to change the phenotype of neighboring cells [109,128]. 

## 11. Exosomes and Macrophages

In both human and murine models, exosomes released by B lymphocytes have the capacity to stimulate specific CD4+ T cell clones in vitro, suggesting a possible role of exosomes as vehicles for major histocompatibility complex (MHC) class II—peptide complexes between immune system cells. These potential roles as mediators of immune responses, and the suggestion of a possible use of exosomes as immunotherapeutic agents, has led to a myriad of articles related to the immune function of exosomes in vitro and in vivo [172]. A number of studies indicated that cardiovascular system–related cells, including platelets, erythrocytes, endothelial cells, leukocytes, monocytes, macrophages, and smooth muscle cells, release EVs that play biological and/or pathological roles in cardiovascular disease, primarily by altering immune function [173,174,175]. 

Macrophage-derived exosomes represent a large portion of the circulating microvesicles in blood [176]. Exosomes from cells infected with intracellular pathogens stimulate a Toll-like receptor-dependent inflammatory response in recipient cells, while dendritic cell (DC)-derived exosomes suppress the onset of murine collagen-induced arthritis and reduce its severity [177,178]. It is also reported that inflammatory stroke can induce extracellular vesicles macrophage activation [179], and these EVs can act as messengers of macrophages sensing atherogenic stimuli [180]. Furthermore, endothelial EVs can modulate the macrophage phenotype with potential implications to atherosclerosis in patients [181]. Conversely, macrophage-derived exosomes induce inflammatory factors in endothelial cells under hypertensive conditions [182], while macrophage-secreted exosomes can deliver an miRNA-21 inhibitor to regulate BGC-823 cell proliferation of gastric cancer cells [183]. 

Exosomes released from monocytes/macrophages can exert several different functions. For example, these exosomes were shown to cause inflammation-induced programmed cell death in vascular smooth muscle cells via transfer of functional pyroptotic caspase-1 [184]. Macrophage-derived exosomes induced differentiation of naïve monocyte recipient cells to macrophages [185]. The macrophage-derived vesicles contained high levels of the miRNA molecule miR-223, which is an important regulator of myeloid cell proliferation and differentiation. In addition, EVs released by macrophages contain MHC class II and costimulatory molecules, and similar to DC-derived EVs, can play a role in antigen presentation [186,187].

## 12. Exosomes and Metabolic Dysfunction

Circulating exosomes have been linked to macrovascular and microvascular dysfunction in human metabolic syndrome and diabetes. For example, patients with metabolic diseases, in particular insulin resistance and type 2 diabetes mellitus, are likely to develop cardiovascular disease including atherosclerosis, stroke, and coronary artery disease [188]. Furthermore, increased EVs are a hallmark of CVD including atherosclerosis, hypertension, and following stroke or myocardial infarction [189,190]. Several studies have examined the role that exosomes play in modulating insulin signaling in mouse models and in vitro studies [191,192]; however, a few studies examine EVs in type 2 diabetes using human cohorts [193]. Clinical studies support the hypothesis that exosomes released from various cell types play roles in the progression of metabolic disorders including type 2 diabetes in both in vitro and in vivo [194]. In type 2 diabetes mellitus (T2DM), exosomes secreted from skeletal muscle [195], vWAT [196], and hepatocytes [197] can transfer both functional proteins and RNA species that regulate the metabolic function of both remote tissues and of adjacent cells.

Exosomes are released by most of the cells into the circulation and bodily fluids display different protein and RNA contents in healthy subjects and patients with different diseases, which can be measured as potential diagnostic markers [198,199]. Several studies have examined circulating exosomes in human obesity and metabolic syndrome [200,201,202,203]. Obesity and other associated metabolic disorders induce increased secretion of vesicles incorporating specific RNAs and proteins as observed in both rodents and humans [204,205]. The current interest in exosomes derives not only from their great potential as novel biomarkers, but also as a new way to deliver innovative therapies to specific target cells. It has been suggested that exosomes possess therapeutic potential through reprogramming of target cells, affording modulation of cellular processes and secretomes—the molecules secreted by cells—and eventually favoring tissue repair after reprogramming of target cells [203]. In addition, there is a possibility that source-cleared exosomes could become a valid marker or therapeutic tool for obesity by decreasing the release of exosomes. Also, these exosomes can be loaded with designed contents (specific proteins, miRNAs, or even chemical medicines) which can be transferred automatically to the target cells to activate intracellular signal pathways to achieve a therapeutic effect [203]. Circulating exosomal miRNAs are currently explored and potentially represent novel biomarkers for metabolic syndrome [206]. For example, Karolina et al. compared exosomes based circulating miRNAs between patients with metabolic syndrome, hypertension, and healthy controls. Results showed that circulating levels of miRNA-17, miRNA-197, miRNA-509-5p, miRNA-92a and miRNA-320a significantly increased in metabolic syndrome patients [207]. The importance of exosomes is now further highlighted by the evidence that they can also be considered as disease biomarkers, as well as possible drug, vaccine, or gene vector delivery tools with potential therapeutic applications [208,209,210,211,212].

Studies in the *db/db* diabetic mouse model demonstrated that exosomes from adipose tissues activate macrophages and promote expression of IL-6 and TNF-α, suggesting that EVs from diabetic mice may convey inflammatory signals [53]. Exosomes derived from insulin resistant mice modulate insulin signaling in skeletal muscle and pancreatic β-cells further suggesting a role for EVs in insulin signaling in mice [191,213]. Recent evidence shows that ATM-derived exosomes may modulate insulin resistance in mice through transfer of specific miRNAs [214]. Exosomes from human adipocyte-explants have been shown to modulate the release of inflammatory cytokines in macrophages [200,215], and as a possible mediator of inflammation and immune crosstalk in diabetic murine models and insulin resistant tissue [53,200,215]. Specifically, adipose tissue-derived exosomes from both mouse and human explants increased IL-6 production in macrophages [200,215]. In addition, two recent studies have reported the presence of an association between exosomes and metabolic dysfunction, and such work could inferentially be adapted to study the impact of OSA on metabolic function. One such study showed that *ob/ob* mice display elevated numbers of exosomes compared to wild-type mice [216]. The second study demonstrated that exosome levels bearing cystatin C were positively related to metabolic complications of obesity in patients with clinically vascular diseases [200]. These findings uncover a potential role of exosomes in the pathogenesis of metabolic diseases. Several other studies have identified exosomes in the culture supernatants of mouse adipose tissues [53], rat primary adipocytes [217], and mouse adipocyte cell line 3T3-L1 [218] that exhibit biological activity. For example, exosomes isolated from the culture supernatant of visceral adipose tissue excised from mice showed that injection of the exosomes derived from diet-induced or genetically (leptin-deficient (*ob/ob*) obese mice into wild-type lean mice results in macrophage activation and insulin resistance [53]. Additionally, isolated exosomes from the supernatants of differentiated 3T3-L1 cells under hypoxic conditions are enriched in enzymes related to lipogenesis and promote lipid accumulation in recipient 3T3-L1 adipocytes [218].

**Figure 4:**
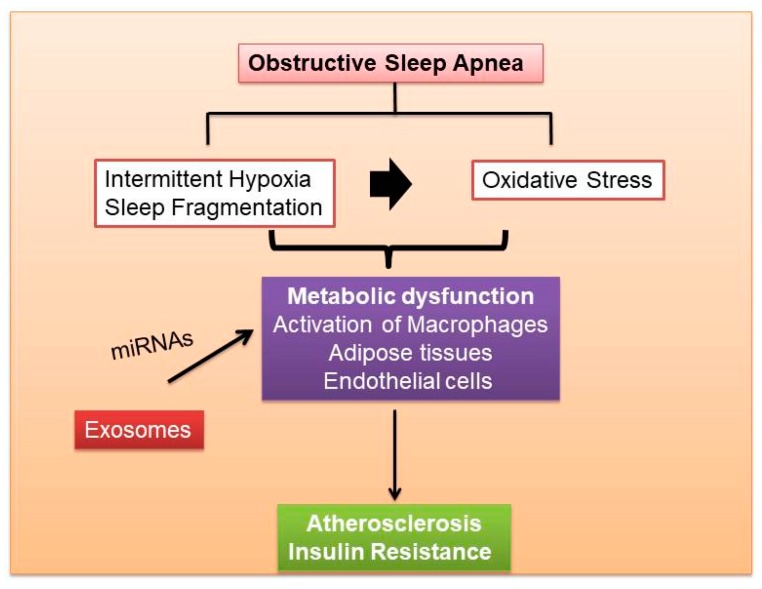
The potential role of exosomes in the metabolic dysfunction and atherosclerosis induced by OSA, and involving interactions among macrophages, adipose tissues and endothelial cells.

The pathogenic mechanisms resulting in monocyte recruitment to adipocytes in OSA and other sleep disorders are under intense investigation and remain incompletely understood. We have recently demonstrated that circulating exosomes from patients suffering from the obesity hypoventilation syndrome (OHS), the most severe form of OSA, or from mice exposed to either long-term IH or SF, promote reduced insulin sensitivity in naïve adipocytes in vitro, and that the exosome cargo biological effects are attenuated when exosomes are obtained after adherent and effective treatment with continuous positive pressure ventilation (CPAP) [11]. Of note, the beneficial effects of CPAP on exosome cargo properties are undetectable among OSA patients who opted not to receive any treatment [11]. EVs have also been studied as potential biomarkers and effector in CVD risk of OSA, and plasma concentrations of exosome subtypes may serve as markers of endothelial dysfunction in CVD [128,219,220,221]. Figure 4 shows the effects of OSA on metabolic dysfunction including endothelial and adipose tissues in relation to atherosclerosis. Under pathological conditions such as IH, exosomes could contribute to the establishment of a pro-inflammatory phenotype that leads to endothelial dysfunction and promotes atherogenesis [222]. In a murine model of persistent pulmonary hypertension, it was shown that exosomes suppressed the infiltration of macrophages and the release of pro-inflammatory and pro-proliferative mediators, including monocyte chemoattractant protein-1 and hypoxia-inducible mitogenic factor. The improvement of microenvironment by exosomes was mediated by miRNAs. MiRNA-204 was inhibited by STAT3 under pathological conditions such as chronic hypoxia, but this inhibitory effect was reversed by exosomes. On the other hand, the pro-proliferative miRNA-17 was induced by hypoxia, but inhibited by exosomes [223]. Furthermore, the effect of exosomes on ischemic reperfusion injury (IR) in mice was reported [224]. In a murine model of persistent pulmonary hypertension, it was shown that exosomes suppressed the infiltration of macrophages and the release of pro-inflammatory and pro-proliferative mediators, including monocyte chemoattractant protein-1 and hypoxia-inducible mitogenic factor. The improvement of microenvironment by exosomes was mediated by miRNAs. MiRNA-204 was inhibited by STAT3 under pathological conditions such as chronic hypoxia, but this inhibitory effect was reversed by exosomes. On the other hand, the pro-proliferative miRNA-17 was induced by hypoxia, but inhibited by exosomes [223]. Furthermore, the effect of exosomes on ischemic reperfusion injury (IR) in mice was reported [224].

## 13. Conclusions

Metabolic homeostasis emerges from the complex, multidirectional crosstalk between key metabolic tissues including adipose tissues, liver, and skeletal muscle. The pathophysiological effects of exosomes and their influence on target cells, depend on the condition in which they are released. In this context, OSA is a highly prevalent disease that carries an independent risk of facilitating metabolic diseases such as atherosclerosis and insulin resistance, ultimately leading to increased overall mortality. 

Exosomes are multifunctional biological entities that are secreted from many mammalian cells and underlie regulation of immune responses and transfer of bioactive molecules between cells. Exosomes are implicated in a growing range of human diseases, including the spectrum of conditions associated with obesity and the metabolic syndrome. In this setting, dysfunction of endothelial cells and recruitment of infiltrating monocytes/macrophages orchestrate complex signaling and communication in the initiation and development of atherosclerosis, and these processes are generally achieved by direct cell-cell contact or transfer of secreted paracrine molecules. However, circulating exosomes containing proteins and nucleic acids can interact with and modify local and distant cellular targets, and as such modulate the initiation and progression of atherosclerosis. Similarly, exosomes from multiple cellular and tissue sources, including macrophages, can foster the development of insulin resistance and metabolic dysfunction, and are emerging as critical mediators of these morbidities in the context of OSA. 

Owing to their ubiquitous presence and stability in various human fluids, and because their contents reflect the characteristics of the parent cell, circulating exosomes, and their constituent miRNAs have been explored as a readily accessible source of novel diagnostic and prognostic biomarkers in metabolic disease, as well as in the prediction of OSA morbid phenotypic expression. 

Taking advantage of exosome properties and their ubiquity, bioengineered exosomes enriched with miRNA, lipids, or drugs may be potentially used in therapy to accurately deliver desirable information to a tissue or cell target. For example, exosomes can mediate the cross talk between adipose tissues and macrophages that facilitates the deregulation of immune and metabolic homeostasis in vWAT, raising the potential opportunity for exosome-based therapeutics in obesity, diabetes, or in OSA-induced metabolic disease. The potential aspirations and ultimately impact of Precision Medicine initiatives warrant a systematic undertaking to develop new methods to interrogate exosomes populations across biological fluids and to foster their therapeutic potential.

## References

[1] Al Lawati N.M., Patel S.R., Ayas N.T. (2009). Epidemiology, risk factors, and consequences of obstructive sleep apnea and short sleep duration. Prog. Cardiovasc. Dis..

[2] Drager L.F., McEvoy R.D., Barbe F., Lorenzi-Filho G., Redline S., Initiative I. (2017). Sleep Apnea and Cardiovascular Disease: Lessons From Recent Trials and Need for Team Science. Circulation.

[3] Koo D.L., Nam H., Thomas R.J., Yun C.H. (2018). Sleep Disturbances as a Risk Factor for Stroke. J. Stroke.

[4] Xie J., Sert Kuniyoshi F.H., Covassin N., Singh P., Gami A.S., Chahal C.A.A., Somers V.K. (2018). Excessive Daytime Sleepiness Independently Predicts Increased Cardiovascular Risk After Myocardial Infarction. J. Am. Heart Assoc..

[5] Mokhlesi B., Ham S.A., Gozal D. (2016). The effect of sex and age on the comorbidity burden of OSA: An observational analysis from a large nationwide US health claims database. Eur. Respir. J..

[6] Tufik S., Santos-Silva R., Taddei J.A., Bittencourt L.R. (2010). Obstructive sleep apnea syndrome in the Sao Paulo Epidemiologic Sleep Study. Sleep Med..

[7] Heinzer R., Vat S., Marques-Vidal P., Marti-Soler H., Andries D., Tobback N., Mooser V., Preisig M., Malhotra A., Waeber G. (2015). Prevalence of sleep-disordered breathing in the general population: The HypnoLaus study. Lancet Respir. Med..

[8] Holley A.B., Phillips B. (2018). The Next 25 Years of Obstructive Sleep Apnea Epidemiology-Don’t Keep Repeating Past Mistakes. Am. J. Respir. Crit. Care Med..

[9] Gileles-Hillel A., Kheirandish-Gozal L., Gozal D. (2016). Biological plausibility linking sleep apnoea and metabolic dysfunction. Nat. Rev. Endocrinol..

[10] Peppard P.E., Young T., Barnet J.H., Palta M., Hagen E.W., Hla K.M. (2013). Increased prevalence of sleep-disordered breathing in adults. Am. J. Epidemiol..

[11] Khalyfa A., Gozal D., Masa J.F., Marin J.M., Qiao Z., Corral J., Gonzalez M., Marti S., Kheirandish-Gozal L., Egea C. (2018). Sleep-disordered breathing, circulating exosomes, and insulin sensitivity in adipocytes. Int. J. Obes..

[12] Poroyko V.A., Carreras A., Khalyfa A., Khalyfa A.A., Leone V., Peris E., Almendros I., Gileles-Hillel A., Qiao Z., Hubert N. (2016). Chronic Sleep Disruption Alters Gut Microbiota, Induces Systemic and Adipose Tissue Inflammation and Insulin Resistance in Mice. Sci. Rep..

[13] Hakim F., Wang Y., Carreras A., Hirotsu C., Zhang J., Peris E., Gozal D. (2015). Chronic sleep fragmentation during the sleep period induces hypothalamic endoplasmic reticulum stress and PTP1b-mediated leptin resistance in male mice. Sleep.

[14] Wang Y., Carreras A., Lee S., Hakim F., Zhang S.X., Nair D., Ye H., Gozal D. (2014). Chronic sleep fragmentation promotes obesity in young adult mice. Obesity.

[15] Gharib S.A., Khalyfa A., Abdelkarim A., Bhushan B., Gozal D. (2012). Integrative miRNA-mRNA profiling of adipose tissue unravels transcriptional circuits induced by sleep fragmentation. PLoS ONE.

[16] Kent B.D., Grote L., Ryan S., Pepin J.L., Bonsignore M.R., Tkacova R., Saaresranta T., Verbraecken J., Levy P., Hedner J. (2014). Diabetes mellitus prevalence and control in sleep-disordered breathing: The European Sleep Apnea Cohort (ESADA) study. Chest.

[17] Perrini S., Cignarelli A., Quaranta V.N., Falcone V.A., Kounaki S., Porro S., Ciavarella A., Ficarella R., Barbaro M., Genchi V.A. (2017). Correction of intermittent hypoxia reduces inflammation in obese subjects with obstructive sleep apnea. JCI Insight.

[18] Louis M., Punjabi N.M. (2009). Effects of acute intermittent hypoxia on glucose metabolism in awake healthy volunteers. J. Appl. Physiol..

[19] Jun J.C., Shin M.K., Devera R., Yao Q., Mesarwi O., Bevans-Fonti S., Polotsky V.Y. (2014). Intermittent hypoxia-induced glucose intolerance is abolished by alpha-adrenergic blockade or adrenal medullectomy. Am. J. Physiol. Endocrinol. Metab..

[20] Polak J., Shimoda L.A., Drager L.F., Undem C., McHugh H., Polotsky V.Y., Punjabi N.M. (2013). Intermittent hypoxia impairs glucose homeostasis in C57BL6/J mice: Partial improvement with cessation of the exposure. Sleep.

[21] Iiyori N., Alonso L.C., Li J., Sanders M.H., Garcia-Ocana A., O’Doherty R.M., Polotsky V.Y., O’Donnell C.P. (2007). Intermittent hypoxia causes insulin resistance in lean mice independent of autonomic activity. Am. J. Respir. Crit. Care Med..

[22] Tripathi A., Melnik A.V., Xue J., Poulsen O., Meehan M.J., Humphrey G., Jiang L., Ackermann G., McDonald D., Zhou D. (2018). Intermittent Hypoxia and Hypercapnia, a Hallmark of Obstructive Sleep Apnea, Alters the Gut Microbiome and Metabolome. mSystems.

[23] Conotte S., Tassin A., Conotte R., Colet J.M., Zouaoui Boudjeltia K., Legrand A. (2018). Metabonomic profiling of chronic intermittent hypoxia in a mouse model. Respir. Physiol. Neurobiol..

[24] Thomas A., Belaidi E., Moulin S., Horman S., van der Zon G.C., Viollet B., Levy P., Bertrand L., Pepin J.L., Godin-Ribuot D. (2017). Chronic Intermittent Hypoxia Impairs Insulin Sensitivity but Improves Whole-Body Glucose Tolerance by Activating Skeletal Muscle AMPK. Diabetes.

[25] Khalyfa A., Qiao Z., Gileles-Hillel A., Khalyfa A.A., Akbarpour M., Popko B., Gozal D. (2017). Activation of the Integrated Stress Response and Metabolic Dysfunction in a Murine Model of Sleep Apnea. Am. J. Respir. Cell Mol. Biol..

[26] Murphy A.M., Thomas A., Crinion S.J., Kent B.D., Tambuwala M.M., Fabre A., Pepin J.L., Roche H.M., Arnaud C., Ryan S. (2017). Intermittent hypoxia in obstructive sleep apnoea mediates insulin resistance through adipose tissue inflammation. Eur. Respir. J..

[27] Ryan S. (2017). Adipose tissue inflammation by intermittent hypoxia: Mechanistic link between obstructive sleep apnoea and metabolic dysfunction. J. Physiol..

[28] Wang Y.C., McPherson K., Marsh T., Gortmaker S.L., Brown M. (2011). Health and economic burden of the projected obesity trends in the USA and the UK. Lancet.

[29] Alkhouri N., Gornicka A., Berk M.P., Thapaliya S., Dixon L.J., Kashyap S., Schauer P.R., Feldstein A.E. (2010). Adipocyte apoptosis, a link between obesity, insulin resistance, and hepatic steatosis. J. Biol. Chem..

[30] Hotamisligil G.S. (2006). Inflammation and metabolic disorders. Nature.

[31] Spruyt K., Gozal D. (2012). A mediation model linking body weight, cognition, and sleep-disordered breathing. Am. J. Respir. Crit. Care Med..

[32] Spruyt K., Molfese D.L., Gozal D. (2011). Sleep duration, sleep regularity, body weight, and metabolic homeostasis in school-aged children. Pediatrics.

[33] Cappuccio F.P., D’Elia L., Strazzullo P., Miller M.A. (2010). Sleep duration and all-cause mortality: A systematic review and meta-analysis of prospective studies. Sleep.

[34] Dean E., Bloom A., Cirillo M., Hong Q., Jawl B., Jukes J., Nijjar M., Sadovich S., Bruno S.S. (2012). Association between habitual sleep duration and blood pressure and clinical implications: A systematic review. Blood Press..

[35] Koren D., Dumin M., Gozal D. (2016). Role of sleep quality in the metabolic syndrome. Diabetes Metab. Syndr. Obes..

[36] Itani O., Kaneita Y., Tokiya M., Jike M., Murata A., Nakagome S., Otsuka Y., Ohida T. (2017). Short sleep duration, shift work, and actual days taken off work are predictive life-style risk factors for new-onset metabolic syndrome: A seven-year cohort study of 40,000 male workers. Sleep Med..

[37] Senaratna C.V., Perret J.L., Lodge C.J., Lowe A.J., Campbell B.E., Matheson M.C., Hamilton G.S., Dharmage S.C. (2017). Prevalence of obstructive sleep apnea in the general population: A systematic review. Sleep Med. Rev..

[38] Carneiro G., Zanella M.T. (2018). Obesity metabolic and hormonal disorders associated with obstructive sleep apnea and their impact on the risk of cardiovascular events. Metabolism.

[39] Bluher M. (2012). Are there still healthy obese patients?. Curr. Opin. Endocrinol. Diabetes Obes..

[40] Engin A.B. (2017). Adipocyte-Macrophage Cross-Talk in Obesity. Adv. Exp. Med. Biol..

[41] Thomas D., Apovian C. (2017). Macrophage functions in lean and obese adipose tissue. Metabolism.

[42] Villarroya F., Cereijo R., Villarroya J., Gavalda-Navarro A., Giralt M. (2018). Toward an Understanding of How Immune Cells Control Brown and Beige Adipobiology. Cell Metab..

[43] Ivanov S., Merlin J., Lee M.K.S., Murphy A.J., Guinamard R.R. (2018). Biology and function of adipose tissue macrophages, dendritic cells and B cells. Atherosclerosis.

[44] Coats B.R., Schoenfelt K.Q., Barbosa-Lorenzi V.C., Peris E., Cui C., Hoffman A., Zhou G., Fernandez S., Zhai L., Hall B.A. (2017). Metabolically Activated Adipose Tissue Macrophages Perform Detrimental and Beneficial Functions during Diet-Induced Obesity. Cell Rep..

[45] Becker L., Liu N.C., Averill M.M., Yuan W., Pamir N., Peng Y., Irwin A.D., Fu X., Bornfeldt K.E., Heinecke J.W. (2012). Unique proteomic signatures distinguish macrophages and dendritic cells. PLoS ONE.

[46] Becker L., Gharib S.A., Irwin A.D., Wijsman E., Vaisar T., Oram J.F., Heinecke J.W. (2010). A macrophage sterol-responsive network linked to atherogenesis. Cell Metab..

[47] Ouchi N., Parker J.L., Lugus J.J., Walsh K. (2011). Adipokines in inflammation and metabolic disease. Nat. Rev. Immunol..

[48] Li C., Xu M.M., Wang K., Adler A.J., Vella A.T., Zhou B. (2018). Macrophage polarization and meta-inflammation. Transl. Res..

[49] Lumeng C.N., Bodzin J.L., Saltiel A.R. (2007). Obesity induces a phenotypic switch in adipose tissue macrophage polarization. J. Clin. Investig..

[50] Nguyen M.T., Favelyukis S., Nguyen A.K., Reichart D., Scott P.A., Jenn A., Liu-Bryan R., Glass C.K., Neels J.G., Olefsky J.M. (2007). A subpopulation of macrophages infiltrates hypertrophic adipose tissue and is activated by free fatty acids via Toll-like receptors 2 and 4 and JNK-dependent pathways. J. Biol. Chem..

[51] Weisberg S.P., McCann D., Desai M., Rosenbaum M., Leibel R.L., Ferrante A.W. (2003). Obesity is associated with macrophage accumulation in adipose tissue. J. Clin. Investig..

[52] Reardon C.A., Lingaraju A., Schoenfelt K.Q., Zhou G., Cui C., Jacobs-El H., Babenko I., Hoofnagle A., Czyz D., Shuman H. (2018). Obesity and Insulin Resistance Promote Atherosclerosis through an IFNgamma-Regulated Macrophage Protein Network. Cell Rep..

[53] Deng Z.B., Poliakov A., Hardy R.W., Clements R., Liu C., Liu Y., Wang J., Xiang X., Zhang S., Zhuang X. (2009). Adipose tissue exosome-like vesicles mediate activation of macrophage-induced insulin resistance. Diabetes.

[54] Ardoin S.P., Shanahan J.C., Pisetsky D.S. (2007). The role of microparticles in inflammation and thrombosis. Scand. J. Immunol..

[55] Camussi G., Deregibus M.C., Bruno S., Cantaluppi V., Biancone L. (2010). Exosomes/microvesicles as a mechanism of cell-to-cell communication. Kidney Int..

[56] Lasser C., Alikhani V.S., Ekstrom K., Eldh M., Paredes P.T., Bossios A., Sjostrand M., Gabrielsson S., Lotvall J., Valadi H. (2011). Human saliva, plasma and breast milk exosomes contain RNA: Uptake by macrophages. J. Transl. Med..

[57] Mosser D.M., Edwards J.P. (2008). Exploring the full spectrum of macrophage activation. Nat. Rev. Immunol..

[58] Guilliams M., Scott C.L. (2017). Does niche competition determine the origin of tissue-resident macrophages?. Nat. Rev. Immunol..

[59] Moore M.A., Metcalf D. (1970). Ontogeny of the haemopoietic system: Yolk sac origin of in vivo and in vitro colony forming cells in the developing mouse embryo. Br. J. Haematol..

[60] Ginhoux F., Guilliams M. (2016). Tissue-Resident Macrophage Ontogeny and Homeostasis. Immunity.

[61] Varol C., Mildner A., Jung S. (2015). Macrophages: Development and tissue specialization. Annu. Rev. Immunol..

[62] Van de Laar L., Saelens W., De Prijck S., Martens L., Scott C.L., Van Isterdael G., Hoffmann E., Beyaert R., Saeys Y., Lambrecht B.N. (2016). Yolk Sac Macrophages, Fetal Liver, and Adult Monocytes Can Colonize an Empty Niche and Develop into Functional Tissue-Resident Macrophages. Immunity.

[63] Perdiguero E.G., Geissmann F. (2016). The development and maintenance of resident macrophages. Nat. Immunol..

[64] Pittet M.J., Nahrendorf M., Swirski F.K. (2014). The journey from stem cell to macrophage. Ann. N. Y. Acad. Sci..

[65] Scott C.L., Zheng F., De Baetselier P., Martens L., Saeys Y., De Prijck S., Lippens S., Abels C., Schoonooghe S., Raes G. (2016). Bone marrow-derived monocytes give rise to self-renewing and fully differentiated Kupffer cells. Nat. Commun..

[66] Eguiluz-Gracia I., Schultz H.H., Sikkeland L.I., Danilova E., Holm A.M., Pronk C.J., Agace W.W., Iversen M., Andersen C., Jahnsen F.L. (2016). Long-term persistence of human donor alveolar macrophages in lung transplant recipients. Thorax.

[67] Mass E., Ballesteros I., Farlik M., Halbritter F., Gunther P., Crozet L., Jacome-Galarza C.E., Handler K., Klughammer J., Kobayashi Y. (2016). Specification of tissue-resident macrophages during organogenesis. Science.

[68] Davies L.C., Jenkins S.J., Allen J.E., Taylor P.R. (2013). Tissue-resident macrophages. Nat. Immunol..

[69] Dey A., Allen J., Hankey-Giblin P.A. (2014). Ontogeny and polarization of macrophages in inflammation: Blood monocytes versus tissue macrophages. Front. Immunol..

[70] Sheng J., Ruedl C., Karjalainen K. (2015). Most Tissue-Resident Macrophages Except Microglia Are Derived from Fetal Hematopoietic Stem Cells. Immunity.

[71] Epelman S., Lavine K.J., Randolph G.J. (2014). Origin and functions of tissue macrophages. Immunity.

[72] Hashimoto D., Chow A., Noizat C., Teo P., Beasley M.B., Leboeuf M., Becker C.D., See P., Price J., Lucas D. (2013). Tissue-resident macrophages self-maintain locally throughout adult life with minimal contribution from circulating monocytes. Immunity.

[73] Perdiguero E.G., Klapproth K., Schulz C., Busch K., de Bruijn M., Rodewald H.R., Geissmann F. (2015). The Origin of Tissue-Resident Macrophages: When an Erythro-myeloid Progenitor Is an Erythro-myeloid Progenitor. Immunity.

[74] Fogg D.K., Sibon C., Miled C., Jung S., Aucouturier P., Littman D.R., Cumano A., Geissmann F. (2006). A clonogenic bone marrow progenitor specific for macrophages and dendritic cells. Science.

[75] Gordon S., Taylor P.R. (2005). Monocyte and macrophage heterogeneity. Nat. Rev. Immunol..

[76] Nahrendorf M., Swirski F.K., Aikawa E., Stangenberg L., Wurdinger T., Figueiredo J.L., Libby P., Weissleder R., Pittet M.J. (2007). The healing myocardium sequentially mobilizes two monocyte subsets with divergent and complementary functions. J. Exp. Med..

[77] Sunderkotter C., Nikolic T., Dillon M.J., Van Rooijen N., Stehling M., Drevets D.A., Leenen P.J. (2004). Subpopulations of mouse blood monocytes differ in maturation stage and inflammatory response. J. Immunol..

[78] Geissmann F., Jung S., Littman D.R. (2003). Blood monocytes consist of two principal subsets with distinct migratory properties. Immunity.

[79] Parisi L., Gini E., Baci D., Tremolati M., Fanuli M., Bassani B., Farronato G., Bruno A., Mortara L. (2018). Macrophage Polarization in Chronic Inflammatory Diseases: Killers or Builders?. J. Immunol. Res..

[80] Van den Bossche J., Saraber D.L. (2018). Metabolic regulation of macrophages in tissues. Cell Immunol..

[81] Gordon S. (2007). The macrophage: Past, present and future. Eur. J. Immunol..

[82] Chanmee T., Ontong P., Konno K., Itano N. (2014). Tumor-associated macrophages as major players in the tumor microenvironment. Cancers.

[83] Ouedraogo R., Daumas A., Ghigo E., Capo C., Mege J.L., Textoris J. (2012). Whole-cell MALDI-TOF MS: A new tool to assess the multifaceted activation of macrophages. J. Proteom..

[84] Funes S.C., Rios M., Escobar-Vera J., Kalergis A.M. (2018). Implications of macrophage polarization in autoimmunity. Immunology.

[85] Franken L., Schiwon M., Kurts C. (2016). Macrophages: Sentinels and regulators of the immune system. Cell Microbiol..

[86] Dantzer R., Capuron L., Irwin M.R., Miller A.H., Ollat H., Perry V.H., Rousey S., Yirmiya R. (2008). Identification and treatment of symptoms associated with inflammation in medically ill patients. Psychoneuroendocrinology.

[87] Condeelis J., Pollard J.W. (2006). Macrophages: Obligate partners for tumor cell migration, invasion, and metastasis. Cell.

[88] Biswas S.K., Gangi L., Paul S., Schioppa T., Saccani A., Sironi M., Bottazzi B., Doni A., Vincenzo B., Pasqualini F. (2006). A distinct and unique transcriptional program expressed by tumor-associated macrophages (defective NF-Kappab and enhanced IRF-3/STAT1 activation). Blood.

[89] Beattie L., Sawtell A., Mann J., Frame T.C.M., Teal B., de Labastida Rivera F., Brown N., Walwyn-Brown K., Moore J.W.J., MacDonald S. (2016). Bone marrow-derived and resident liver macrophages display unique transcriptomic signatures but similar biological functions. J. Hepatol..

[90] Gautier E.L., Shay T., Miller J., Greter M., Jakubzick C., Ivanov S., Helft J., Chow A., Elpek K.G., Gordonov S. (2012). Gene-expression profiles and transcriptional regulatory pathways that underlie the identity and diversity of mouse tissue macrophages. Nat. Immunol..

[91] Gibbings S.L., Goyal R., Desch A.N., Leach S.M., Prabagar M., Atif S.M., Bratton D.L., Janssen W., Jakubzick C.V. (2015). Transcriptome analysis highlights the conserved difference between embryonic and postnatal-derived alveolar macrophages. Blood.

[92] Italiani P., Boraschi D. (2014). From Monocytes to M1/M2 Macrophages: Phenotypical vs. Functional Differentiation. Front. Immunol..

[93] Grivennikov S.I., Greten F.R., Karin M. (2010). Immunity, inflammation, and cancer. Cell.

[94] Suganami T., Ogawa Y. (2010). Adipose tissue macrophages: Their role in adipose tissue remodeling. J. Leukoc. Biol..

[95] Sica A., Larghi P., Mancino A., Rubino L., Porta C., Totaro M.G., Rimoldi M., Biswas S.K., Allavena P., Mantovani A. (2008). Macrophage polarization in tumour progression. Semin. Cancer Biol..

[96] Duluc D., Corvaisier M., Blanchard S., Catala L., Descamps P., Gamelin E., Ponsoda S., Delneste Y., Hebbar M., Jeannin P. (2009). Interferon-gamma reverses the immunosuppressive and protumoral properties and prevents the generation of human tumor-associated macrophages. Int. J. Cancer.

[97] Roszer T. (2015). Understanding the Mysterious M2 Macrophage through Activation Markers and Effector Mechanisms. Mediat. Inflamm..

[98] Haney M.J., Klyachko N.L., Zhao Y., Gupta R., Plotnikova E.G., He Z., Patel T., Piroyan A., Sokolsky M., Kabanov A.V. (2015). Exosomes as drug delivery vehicles for Parkinson’s disease therapy. J. Control. Release.

[99] Fain J.N., Bahouth S.W., Madan A.K. (2004). TNFalpha release by the nonfat cells of human adipose tissue. Int. J. Obes. Relat. Metab. Disord..

[100] Wellen K.E., Hotamisligil G.S. (2005). Inflammation, stress, and diabetes. J. Clin. Investig..

[101] Amano S.U., Cohen J.L., Vangala P., Tencerova M., Nicoloro S.M., Yawe J.C., Shen Y., Czech M.P., Aouadi M. (2014). Local proliferation of macrophages contributes to obesity-associated adipose tissue inflammation. Cell Metab..

[102] Nagareddy P.R., Kraakman M., Masters S.L., Stirzaker R.A., Gorman D.J., Grant R.W., Dragoljevic D., Hong E.S., Abdel-Latif A., Smyth S.S. (2014). Adipose tissue macrophages promote myelopoiesis and monocytosis in obesity. Cell Metab..

[103] Reilly S.M., Saltiel A.R. (2017). Adapting to obesity with adipose tissue inflammation. Nat. Rev. Endocrinol..

[104] Morris D.L., Singer K., Lumeng C.N. (2011). Adipose tissue macrophages: Phenotypic plasticity and diversity in lean and obese states. Curr. Opin. Clin. Nutr. Metab. Care.

[105] Kumari M., Heeren J., Scheja L. (2018). Regulation of immunometabolism in adipose tissue. Semin. Immunopathol..

[106] Muir L.A., Kiridena S., Griffin C., DelProposto J.B., Geletka L., Martinez-Santibanez G., Zamarron B.F., Lucas H., Singer K., O’Rourke R.W. (2018). Rapid adipose tissue expansion triggers unique proliferation and lipid accumulation profiles in adipose tissue macrophages. J. Leukoc. Biol..

[107] Tanaka M., Itoh M., Ogawa Y., Suganami T., Itoh M., Ogawa Y., Suganami T. (2018). Molecular mechanism of obesity-induced ‘metabolic’ tissue remodeling. J. Diabetes Investig..

[108] Wieser V., Adolph T.E., Grander C., Grabherr F., Enrich B., Moser P., Moschen A.R., Kaser S., Tilg H. (2018). Adipose type I interferon signalling protects against metabolic dysfunction. Gut.

[109] Khalyfa A., Kheirandish-Gozal L., Gozal D. (2017). Circulating exosomes in obstructive sleep apnea as phenotypic biomarkers and mechanistic messengers of end-organ morbidity. Respir. Physiol. Neurobiol..

[110] Light M., McCowen K., Malhotra A., Mesarwi O.A. (2017). Sleep apnea, metabolic disease, and the cutting edge of therapy. Metabolism.

[111] Lombardi C., Tobaldini E., Montano N., Losurdo A., Parati G. (2017). Obstructive Sleep Apnea Syndrome (OSAS) and Cardiovascular System. Med. Lav..

[112] Gozal D., Khalyfa A., Qiao Z., Akbarpour M., Maccari R., Ottana R. (2017). Protein-Tyrosine Phosphatase-1B Mediates Sleep Fragmentation-Induced Insulin Resistance and Visceral Adipose Tissue Inflammation in Mice. Sleep.

[113] Khalyfa A., Wang Y., Zhang S.X., Qiao Z., Abdelkarim A., Gozal D. (2014). Sleep fragmentation in mice induces nicotinamide adenine dinucleotide phosphate oxidase 2-dependent mobilization, proliferation, and differentiation of adipocyte progenitors in visceral white adipose tissue. Sleep.

[114] Zhang S.X., Khalyfa A., Wang Y., Carreras A., Hakim F., Neel B.A., Brady M.J., Qiao Z., Hirotsu C., Gozal D. (2014). Sleep fragmentation promotes NADPH oxidase 2-mediated adipose tissue inflammation leading to insulin resistance in mice. Int. J. Obes..

[115] Carreras A., Zhang S.X., Almendros I., Wang Y., Peris E., Qiao Z., Gozal D. (2015). Resveratrol attenuates intermittent hypoxia-induced macrophage migration to visceral white adipose tissue and insulin resistance in male mice. Endocrinology.

[116] Gozal D., Gileles-Hillel A., Cortese R., Li Y., Almendros I., Qiao Z., Khalyfa A.A., Andrade J., Khalyfa A. (2017). Visceral White Adipose Tissue Following Chronic Intermittent and Sustained Hypoxia in Mice. Am. J. Respir. Cell Mol. Biol..

[117] Chawla A., Nguyen K.D., YGoh P. (2011). Macrophage-mediated inflammation in metabolic disease. Nat. Rev. Immunol..

[118] Shoelson S.E., Lee J., Goldfine A.B. (2006). Inflammation and insulin resistance. J. Clin. Investig..

[119] Koppaka S., Kehlenbrink S., Carey M., Li W., Sanchez E., Lee D.E., Lee H., Chen J., Carrasco E., Kishore P. (2013). Reduced adipose tissue macrophage content is associated with improved insulin sensitivity in thiazolidinedione-treated diabetic humans. Diabetes.

[120] Menghini R., Casagrande V., Menini S., Marino A., Marzano V., Hribal M.L., Gentileschi P., Lauro D., Schillaci O., Pugliese G. (2012). TIMP3 overexpression in macrophages protects from insulin resistance, adipose inflammation, and nonalcoholic fatty liver disease in mice. Diabetes.

[121] Xu H., Barnes G.T., Yang Q., Tan G., Yang D., Chou C.J., Sole J., Nichols A., Ross J.S., Tartaglia L.A. (2003). Chronic inflammation in fat plays a crucial role in the development of obesity-related insulin resistance. J. Clin. Investig..

[122] Wellen K.E., Hotamisligil G.S. (2003). Obesity-induced inflammatory changes in adipose tissue. J. Clin. Investig..

[123] Weber C., Noels H. (2011). Atherosclerosis: Current pathogenesis and therapeutic options. Nat. Med..

[124] Chinetti-Gbaguidi G., Colin S., Staels B. (2015). Macrophage subsets in atherosclerosis. Nat. Rev. Cardiol..

[125] De Gaetano M., Crean D., Barry M., Belton O. (2016). M1- and M2-Type Macrophage Responses Are Predictive of Adverse Outcomes in Human Atherosclerosis. Front. Immunol..

[126] Moore K.J., Sheedy F.J., Fisher E.A. (2013). Macrophages in atherosclerosis: A dynamic balance. Nat. Rev. Immunol..

[127] Khalyfa A., Khalyfa A.A., Akbarpour M., Connes P., Romana M., Laping-Carr G., Zhang C., Andrade J., Gozal D. (2016). Extracellular microvesicle microRNAs in children with sickle cell anemia with divergent clinical phenotypes. Br. J. Haematol..

[128] Khalyfa A., Kheirandish-Gozal L., Khalyfa A.A., Philby M.F., Alonso-Alvarez M.L., Bhattacharjee R., Teran Santos J., Huang L., Andrade J., Gozal D. (2016). Circulating plasma extracellular microvesicle miRNA cargo and endothelial dysfunction in OSA children. Am. J. Resp. Crit. Care Med..

[129] Thery C., Ostrowski M., Segura E. (2009). Membrane vesicles as conveyors of immune responses. Nat. Rev. Immunol..

[130] Mathivanan S., Ji H., Simpson R.J. (2010). Exosomes: Extracellular organelles important in intercellular communication. J. Proteom..

[131] Vlassov A.V., Magdaleno S., Setterquist R., Conrad R. (2012). Exosomes: Current knowledge of their composition, biological functions, and diagnostic and therapeutic potentials. Biochim. Biophys. Acta.

[132] Denzer K., Kleijmeer M.J., Heijnen H.F., Stoorvogel W., Geuze H.J. (2000). Exosome: From internal vesicle of the multivesicular body to intercellular signaling device. J. Cell Sci..

[133] Raposo G., Nijman H.W., Stoorvogel W., Liejendekker R., Harding C.V., Melief C.J., Geuze H.J. (1996). B lymphocytes secrete antigen-presenting vesicles. J. Exp. Med..

[134] Dragovic R.A., Gardiner C., Brooks A.S., Tannetta D.S., Ferguson D.J., Hole P., Carr B., Redman C.W., Harris A.L., Dobson P.J. (2011). Sizing and phenotyping of cellular vesicles using Nanoparticle Tracking Analysis. Nanomedicine.

[135] Keerthikumar S., Chisanga D., Ariyaratne D., Al Saffar H., Anand S., Zhao K., Samuel M., Pathan M., Jois M., Chilamkurti N. (2016). ExoCarta: A Web-Based Compendium of Exosomal Cargo. J. Mol. Biol..

[136] De Gassart A., Geminard C., Fevrier B., Raposo G., Vidal M. (2003). Lipid raft-associated protein sorting in exosomes. Blood.

[137] Kajimoto T., Okada T., Miya S., Zhang L., Nakamura S. (2013). Ongoing activation of sphingosine 1-phosphate receptors mediates maturation of exosomal multivesicular endosomes. Nat. Commun..

[138] Gezer U., Ozgur E., Cetinkaya M., Isin M., Dalay N. (2014). Long non-coding RNAs with low expression levels in cells are enriched in secreted exosomes. Cell Biol. Int..

[139] Kogure T., Lin W.L., Yan I.K., Braconi C., Patel T. (2011). Intercellular nanovesicle-mediated microRNA transfer: A mechanism of environmental modulation of hepatocellular cancer cell growth. Hepatology.

[140] Valadi H., Ekstrom K., Bossios A., Sjostrand M., Lee J.J., Lotvall J.O. (2007). Exosome-mediated transfer of mRNAs and microRNAs is a novel mechanism of genetic exchange between cells. Nat. Cell Biol..

[141] Mittelbrunn M., Gutierrez-Vazquez C., Villarroya-Beltri C., Gonzalez S., Sanchez-Cabo F., Gonzalez M.A., Bernad A., Sanchez-Madrid F. (2011). Unidirectional transfer of microRNA-loaded exosomes from T cells to antigen-presenting cells. Nat. Commun..

[142] Lee G.H., Lee S.A., No S.K., Lee S.M., Ryu J.Y., Jo K.D., Kwon J.H., Kim O.J., Park H., Kwon O.Y. (2016). Factors contributing to the development of perceived stigma in people with newly diagnosed epilepsy: A one-year longitudinal study. Epilepsy Behav..

[143] Salido-Guadarrama I., Romero-Cordoba S., Peralta-Zaragoza O., Hidalgo-Miranda A., Rodriguez-Dorantes M. (2014). MicroRNAs transported by exosomes in body fluids as mediators of intercellular communication in cancer. Onco Targets Ther..

[144] Khalyfa A., Kheirandish-Gozal L., Bhattacharjee R., Khalyfa A.A., Gozal D. (2016). Circulating microRNAs as Potential Biomarkers of Endothelial Dysfunction in Obese Children. Chest.

[145] Khalyfa A., Zhang C., Khalyfa A.A., Foster G.E., Beaudin A.E., Andrade J., Hanly P.J., Poulin M.J., Gozal D. (2016). Effect on Intermittent Hypoxia on Plasma Exosomal Micro RNA Signature and Endothelial Function in Healthy Adults. Sleep.

[146] Christianson H.C., Svensson K.J., van Kuppevelt T.H., Li J.P., Belting M. (2013). Cancer cell exosomes depend on cell-surface heparan sulfate proteoglycans for their internalization and functional activity. Proc. Natl. Acad. Sci. USA.

[147] Gajos-Michniewicz A., Duechler M., Czyz M. (2014). MiRNA in melanoma-derived exosomes. Cancer Lett..

[148] Tian T., Zhu Y.L., Zhou Y.Y., Liang G.F., Wang Y.Y., Hu F.H., Xiao Z.D. (2014). Exosome uptake through clathrin-mediated endocytosis and macropinocytosis and mediating miR-21 delivery. J. Biol. Chem..

[149] Record M., Carayon K., Poirot M., Silvente-Poirot S. (2014). Exosomes as new vesicular lipid transporters involved in cell-cell communication and various pathophysiologies. Biochim. Biophys. Acta.

[150] Chen C., Zong S., Wang Z., Lu J., Zhu D., Zhang Y., Zhang R., Cui Y. (2018). Visualization and intracellular dynamic tracking of exosomes and exosomal miRNAs using single molecule localization microscopy. Nanoscale.

[151] Horibe S., Tanahashi T., Kawauchi S., Murakami Y., Rikitake Y. (2018). Mechanism of recipient cell-dependent differences in exosome uptake. BMC Cancer.

[152] Khalyfa A., Youssefnia N., Foster G.E., Beaudin A.E., Qiao Z., Pialoux V., Pun M., Hanly P.J., Kheirandish-Gozal L., Poulin M.J. (2017). Plasma Exosomes and Improvements in Endothelial Function by Angiotensin 2 Type 1 Receptor or Cyclooxygenase 2 Blockade following Intermittent Hypoxia. Front. Neurol..

[153] Puzar Dominkus P., Stenovec M., Sitar S., Lasic E., Zorec R., Plemenitas A., Zagar E., Kreft M., Lenassi M. (2018). PKH26 labeling of extracellular vesicles: Characterization and cellular internalization of contaminating PKH26 nanoparticles. Biochim. Biophys. Acta.

[154] Khatri M., Richardson L.A., Meulia T. (2018). Mesenchymal stem cell-derived extracellular vesicles attenuate influenza virus-induced acute lung injury in a pig model. Stem Cell Res. Ther..

[155] Pasalic L., Siupa A., Campbell H., Henderson M.J., Chen V.M.Y. (2016). Enumeration of extracellular vesicles by a new improved flow cytometric method is comparable to fluorescence mode nanoparticle tracking analysis. Nanomedicine.

[156] Stremersch S., Brans T., Braeckmans K., De Smedt S., Raemdonck K. (2017). Nucleic acid loading and fluorescent labeling of isolated extracellular vesicles requires adequate purification. Int. J. Pharm..

[157] Escrevente C., Altevogt P., Costa J. (2011). Interaction and uptake of exosomes by ovarian cancer cells. BMC Cancer.

[158] Lima L.G., Monteiro R.Q., Moreira M.E., Barcinski M.A. (2009). Tumor-derived microvesicles modulate the establishment of metastatic melanoma in a phosphatidylserine-dependent manner. Cancer Lett..

[159] Feng D., Zhao W.L., Ye Y.Y., Bai X.C., Liu R.Q., Chang L.F., Zhou Q., Sui S.F. (2010). Cellular internalization of exosomes occurs through phagocytosis. Traffic.

[160] Nakase I., Futaki S. (2015). Combined treatment with a pH-sensitive fusogenic peptide and cationic lipids achieves enhanced cytosolic delivery of exosomes. Sci. Rep..

[161] Atai N.A., Balaj L., van Veen H., Breakefield X.O., Jarzyna P.A., Van Noorden C.J., Skog J., Maguire C.A. (2013). Heparin blocks transfer of extracellular vesicles between donor and recipient cells. J. Neurooncol..

[162] Bobrie A., Balaj L., van Veen H., Breakefield X.O., Jarzyna P.A., Van Noorden C.J., Skog J., Maguire C.A. (2011). Exosome secretion: Molecular mechanisms and roles in immune responses. Traffic.

[163] Azmi A.S., Bao B., Sarkar F.H. (2013). Exosomes in cancer development, metastasis, and drug resistance: A comprehensive review. Cancer Metastasis Rev..

[164] Pan B.T., Teng K., Wu C., Adam M., Johnstone R.M. (1985). Electron microscopic evidence for externalization of the transferrin receptor in vesicular form in sheep reticulocytes. J. Cell Biol..

[165] Admyre C., Johansson S.M., Qazi K.R., Filen J.J., Lahesmaa R., Norman M., Neve E.P., Scheynius A., Gabrielsson S. (2007). Exosomes with immune modulatory features are present in human breast milk. J. Immunol..

[166] Li P., Kaslan M., Lee S.H., Yao J., Gao Z. (2017). Progress in Exosome Isolation Techniques. Theranostics.

[167] Rak J., Guha A. (2012). Extracellular vesicles--vehicles that spread cancer genes. Bioessays.

[168] Ibrahim A., Marban E. (2016). Exosomes: Fundamental Biology and Roles in Cardiovascular Physiology. Annu. Rev. Physiol..

[169] Mizrak A., Bolukbasi M.F., Ozdener G.B., Brenner G.J., Madlener S., Erkan E.P., Strobel T., Breakefield X.O., Saydam O. (2013). Genetically engineered microvesicles carrying suicide mRNA/protein inhibit schwannoma tumor growth. Mol. Ther..

[170] Choudhuri K., Llodra J., Roth E.W., Tsai J., Gordo S., Wucherpfennig K.W., Kam L.C., Stokes D.L., Dustin M.L. (2014). Polarized release of T-cell-receptor-enriched microvesicles at the immunological synapse. Nature.

[171] Quesenberry P.J., Goldberg L.R., Aliotta J.M., Dooner M.S., Pereira M.G., Wen S., Camussi G. (2014). Cellular phenotype and extracellular vesicles: Basic and clinical considerations. Stem Cells Dev..

[172] Chaput N., Thery C. (2011). Exosomes: Immune properties and potential clinical implementations. Semin. Immunopathol..

[173] Helal O., Thery C. (2011). Increased levels of microparticles originating from endothelial cells, platelets and erythrocytes in subjects with metabolic syndrome: Relationship with oxidative stress. Nutr. Metab. Cardiovasc. Dis..

[174] Rautou P.E., Vion A.C., Amabile N., Chironi G., Simon A., Tedgui A., Boulanger C.M. (2011). Microparticles, vascular function, and atherothrombosis. Circ. Res..

[175] Sarlon-Bartoli G., Bennis Y., Lacroix R., Piercecchi-Marti M.D., Bartoli M.A., Arnaud L., Mancini J., Boudes A., Sarlon E., Thevenin B. (2013). Plasmatic level of leukocyte-derived microparticles is associated with unstable plaque in asymptomatic patients with high-grade carotid stenosis. J. Am. Coll. Cardiol..

[176] Ismail N., Wang Y., Dakhlallah D., Moldovan L., Agarwal K., Batte K., Shah P., Wisler J., Eubank T.D., Tridandapani S. (2013). Macrophage microvesicles induce macrophage differentiation and miR-223 transfer. Blood.

[177] Fabbri M., Paone A., Calore F., Galli R., Croce C.M. (2013). A new role for microRNAs, as ligands of Toll-like receptors. RNA Biol..

[178] McDonald M.K., Tian Y., Qureshi R.A., Gormley M., Ertel A., Gao R., Aradillas Lopez E., Alexander G.M., Sacan A., Fortina P. (2014). Functional significance of macrophage-derived exosomes in inflammation and pain. Pain.

[179] Couch Y., Akbar N., Davis S., Fischer R., Dickens A.M., Neuhaus A.A., Burgess A.I., Rothwell P.M., Buchan A.M. (2017). Inflammatory Stroke Extracellular Vesicles Induce Macrophage Activation. Stroke.

[180] Afonyushkin T., Binder C.J. (2018). Extracellular Vesicles Act as Messengers of Macrophages Sensing Atherogenic Stimuli. Arterioscler. Thromb. Vasc. Biol..

[181] He S., Wu C., Xiao J., Li D., Sun Z., Li M. (2018). Endothelial extracellular vesicles modulate the macrophage phenotype: Potential implications in atherosclerosis. Scand. J. Immunol..

[182] Osada-Oka M., Shiota M., Izumi Y., Nishiyama M., Tanaka M., Yamaguchi T., Sakurai E., Miura K., Iwao H. (2017). Macrophage-derived exosomes induce inflammatory factors in endothelial cells under hypertensive conditions. Hypertens. Res..

[183] Wang J.J., Wang Z.Y., Chen R., Xiong J., Yao Y.L., Wu J.H., Li G.X. (2015). Macrophage-secreted Exosomes Delivering miRNA-21 Inhibitor can Regulate BGC-823 Cell Proliferation. Asian Pac. J. Cancer Prev..

[184] Sarkar A., Mitra S., Mehta S., Raices R., Wewers M.D. (2009). Monocyte derived microvesicles deliver a cell death message via encapsulated caspase-1. PLoS ONE.

[185] Xu J.F., Yang G.H., Pan X.H., Zhang S.J., Zhao C., Qiu B.S., Gu H.F., Hong J.F., Cao L., Chen Y. (2014). Altered microRNA expression profile in exosomes during osteogenic differentiation of human bone marrow-derived mesenchymal stem cells. PLoS ONE.

[186] Qu Y., Ramachandra L., Mohr S., Franchi L., Harding C.V., Nunez G., Dubyak G.R. (2009). P2X7 receptor-stimulated secretion of MHC class II-containing exosomes requires the ASC/NLRP3 inflammasome but is independent of caspase-1. J. Immunol..

[187] Teo B.H., Wong S.H. (2010). MHC class II-associated invariant chain (Ii) modulates dendritic cells-derived microvesicles (DCMV)-mediated activation of microglia. Biochem. Biophys. Res. Commun..

[188] Rask-Madsen C., King G.L. (2013). Vascular complications of diabetes: Mechanisms of injury and protective factors. Cell Metab..

[189] Chen Y., Feng B., Li X., Ni Y., Luo Y. (2012). Plasma endothelial microparticles and their correlation with the presence of hypertension and arterial stiffness in patients with type 2 diabetes. J. Clin. Hypertens..

[190] Kim J., Bhattacharjee R., Khalyfa A., Kheirandish-Gozal L., Capdevila O.S., Wang Y., Gozal D. (2012). DNA methylation in inflammatory genes among children with obstructive sleep apnea. Am. J. Respir. Crit. Care Med..

[191] Aswad H., Forterre A., Wiklander O.P., Vial G., Danty-Berger E., Jalabert A., Lamaziere A., Meugnier E., Pesenti S., Ott C. (2014). Exosomes participate in the alteration of muscle homeostasis during lipid-induced insulin resistance in mice. Diabetologia.

[192] Zhang Y., Shi L., Mei H., Zhang J., Zhu Y., Han X., Zhu D. (2015). Inflamed macrophage microvesicles induce insulin resistance in human adipocytes. Nutr. Metab..

[193] Freeman D.W., Noren Hooten N., Eitan E., Green J., Mode N.A., Bodogai M., Zhang Y., Lehrmann E., Zonderman A.B., Biragyn A. (2018). Altered Extracellular Vesicle Concentration, Cargo and Function in Diabetes Mellitus. Diabetes.

[194] Lee M.J., Park D.H., Kang J.H. (2016). Exosomes as the source of biomarkers of metabolic diseases. Ann. Pediatr. Endocrinol. Metab..

[195] Guescini M., Genedani S., Stocchi V., Agnati L.F. (2010). Astrocytes and Glioblastoma cells release exosomes carrying mtDNA. J. Neural Transm..

[196] Aoki N., Jin-no S., Nakagawa Y., Asai N., Arakawa E., Tamura N., Tamura T., Matsuda T. (2007). Identification and characterization of microvesicles secreted by 3T3-L1 adipocytes: Redox- and hormone-dependent induction of milk fat globule-epidermal growth factor 8-associated microvesicles. Endocrinology.

[197] Conde-Vancells J., Rodriguez-Suarez E., Embade N., Gil D., Matthiesen R., Valle M., Elortza F., Lu S.C., Mato J.M., Falcon-Perez J.M. (2008). Characterization and comprehensive proteome profiling of exosomes secreted by hepatocytes. J. Proteom. Res..

[198] Pant S., Hilton H., Burczynski M.E. (2012). The multifaceted exosome: Biogenesis, role in normal and aberrant cellular function, and frontiers for pharmacological and biomarker opportunities. Biochem. Pharmacol..

[199] Revenfeld A.L., Baek R., Nielsen M.H., Stensballe A., Varming K., Jorgensen M. (2014). Diagnostic and prognostic potential of extracellular vesicles in peripheral blood. Clin. Ther..

[200] Kranendonk M.E., de Kleijn D.P., Kalkhoven E., Kanhai D.A., Uiterwaal C.S., van der Graaf Y., Pasterkamp G., Visseren F.L., Group S.S. (2014). Extracellular vesicle markers in relation to obesity and metabolic complications in patients with manifest cardiovascular disease. Cardiovasc. Diabetol..

[201] Martinez M.C., Andriantsitohaina R. (2017). Extracellular Vesicles in Metabolic Syndrome. Circ. Res..

[202] Yao Z.Y., Chen W.B., Shao S.S., Ma S.Z., Yang C.B., Li M.Z., Zhao J.J., Gao L. (2018). Role of exosome-associated microRNA in diagnostic and therapeutic applications to metabolic disorders. J. Zhejiang Univ. Sci. B.

[203] Milbank E., Martinez M.C., Andriantsitohaina R. (2016). Extracellular vesicles: Pharmacological modulators of the peripheral and central signals governing obesity. Pharmacol. Ther..

[204] Stepanian A., Bourguignat L., Hennou S., Coupaye M., Hajage D., Salomon L., Alessi M.C., Msika S., de Prost D. (2013). Microparticle increase in severe obesity: Not related to metabolic syndrome and unchanged after massive weight loss. Obesity.

[205] Ferrante S.C., Nadler E.P., Pillai D.K., Hubal M.J., Wang Z., Wang J.M., Gordish-Dressman H., Koeck E., Sevilla S., Wiles A.A. (2015). Adipocyte-derived exosomal miRNAs: A novel mechanism for obesity-related disease. Pediatr. Res..

[206] O’Neill S., Bohl M., Gregersen S., Hermansen K., O’Driscoll L. (2016). Blood-Based Biomarkers for Metabolic Syndrome. Trends Endocrinol. Metab..

[207] Karolina D.S., Tavintharan S., Armugam A., Sepramaniam S., Pek S.L., Wong M.T., Lim S.C., Sum C.F., Jeyaseelan K. (2012). Circulating miRNA profiles in patients with metabolic syndrome. J. Clin. Endocrinol. Metab..

[208] Corrado C., Raimondo S., Chiesi A., Ciccia F., De Leo G., Alessandro R. (2013). Exosomes as intercellular signaling organelles involved in health and disease: Basic science and clinical applications. Int. J. Mol. Sci..

[209] Emanueli C., Shearn A.I., Angelini G.D., Sahoo S. (2015). Exosomes and exosomal miRNAs in cardiovascular protection and repair. Vascul. Pharmacol..

[210] Mager I., Breakefield X.O., Wood M.J. (2013). Extracellular vesicles: Biology and emerging therapeutic opportunities. Nat. Rev. Drug. Discov..

[211] Zhang B., Yeo R.W., Tan K.H., Lim S.K. (2016). Focus on Extracellular Vesicles: Therapeutic Potential of Stem Cell-Derived Extracellular Vesicles. Int. J. Mol. Sci..

[212] Ren J., He W., Zheng L., Duan H. (2016). From structures to functions: Insights into exosomes as promising drug delivery vehicles. Biomater. Sci..

[213] Jalabert A., Vial G., Guay C., Wiklander O.P., Nordin J.Z., Aswad H., Forterre A., Meugnier E., Pesenti S., Regazzi R. (2016). Exosome-like vesicles released from lipid-induced insulin-resistant muscles modulate gene expression and proliferation of beta recipient cells in mice. Diabetologia.

[214] Ying W., Riopel M., Bandyopadhyay G., Dong Y., Birmingham A., Seo J.B., Ofrecio J.M., Wollam J., Hernandez-Carretero A., Fu W. (2017). Adipose Tissue Macrophage-Derived Exosomal miRNAs Can Modulate In Vivo and In Vitro Insulin Sensitivity. Cell.

[215] Kranendonk M.E., Visseren F.L., van Balkom B.W., Nolte-’t Hoen E.N., van Herwaarden J.A., de Jager W., Schipper H.S., Brenkman A.B., Verhaar M.C., Wauben M.H. (2014). Human adipocyte extracellular vesicles in reciprocal signaling between adipocytes and macrophages. Obesity.

[216] Phoonsawat W., Aoki-Yoshida A., Tsuruta T., Sonoyama K. (2014). Adiponectin is partially associated with exosomes in mouse serum. Biochem. Biophys. Res. Commun..

[217] Muller G., Jung C., Straub J., Wied S., Kramer W. (2009). Induced release of membrane vesicles from rat adipocytes containing glycosylphosphatidylinositol-anchored microdomain and lipid droplet signalling proteins. Cell Signal.

[218] Sano S., Izumi Y., Yamaguchi T., Yamazaki T., Tanaka M., Shiota M., Osada-Oka M., Nakamura Y., Wei M., Wanibuchi H. (2014). Lipid synthesis is promoted by hypoxic adipocyte-derived exosomes in 3T3-L1 cells. Biochem. Biophys. Res. Commun..

[219] Jansen F., Nickenig G., Werner N. (2017). Extracellular Vesicles in Cardiovascular Disease: Potential Applications in Diagnosis, Prognosis, and Epidemiology. Circ. Res..

[220] Van Niel G., Porto-Carreiro I., Simoes S., Raposo G. (2006). Exosomes: A common pathway for a specialized function. J. Biochem..

[221] Bhattacharjee R., Khalyfa A., Khalyfa A.A., Mokhlesi B., Kheirandish-Gozal L., Almendros I., Peris E., Malhotra A., Gozal D. (2018). Exosomal Cargo Properties, Endothelial Function and Treatment of Obesity Hypoventilation Syndrome: A Proof of Concept Study. J. Clin. Sleep Med..

[222] Agouni A., Lagrue-Lak-Hal A.H., Ducluzeau P.H., Mostefai H.A., Draunet-Busson C., Leftheriotis G., Heymes C., Martinez M.C., Andriantsitohaina R. (2008). Endothelial dysfunction caused by circulating microparticles from patients with metabolic syndrome. Am. J. Pathol..

[223] Lee C., Mitsialis S.A., Aslam M., Vitali S.H., Vergadi E., Konstantinou G., Sdrimas K., Fernandez-Gonzalez A., Kourembanas S. (2012). Exosomes mediate the cytoprotective action of mesenchymal stromal cells on hypoxia-induced pulmonary hypertension. Circulation.

[224] Kalani A., Chaturvedi P., Kamat P.K., Maldonado C., Bauer P., Joshua I.G., Tyagi S.C., Tyagi N. (2016). Curcumin-loaded embryonic stem cell exosomes restored neurovascular unit following ischemia-reperfusion injury. Int. J. Biochem. Cell Biol..

